# Bioactive Protecting Coating of Guar Gum with Thyme Oil to Extend Shelf Life of Tilapia (*Oreoschromis niloticus*) Fillets

**DOI:** 10.3390/polym12123019

**Published:** 2020-12-17

**Authors:** Xochitl Ruelas-Chacon, Alfredo Aguilar-González, María de la Luz Reyes-Vega, René Darío Peralta-Rodríguez, José Corona-Flores, Oscar Noé Rebolloso-Padilla, Antonio Francisco Aguilera-Carbo

**Affiliations:** 1 Department of Food Science and Technology, Autonomous Agrarian University Antonio Narro, Calzada Antonio Narro 1923, Saltillo 25315, Mexico; aguilar2742@hotmail.com; 2 Department of Research and Graduate Studies, Autonomous University of Queretaro, Cerro de las Campanas, Downtown Queretaro 76010, Mexico; luzrega@icloud.com; 3 Department of Polymerization Processes, Research Center for Applied Chemistry, Blvd. Enrique Reyna Hermosillo No. 140, Saltillo 25253, Mexico; rene.peralta@ciqa.edu.mx; 4 Department of Planning, Autonomous Agrarian University Antonio Narro, Calzada Antonio Narro 1923, Saltillo 25315, Mexico; josedaniel.corona@gmail.com; 5 Departament of Animal Production, Autonomous Agrarian University Antonio Narro, Calzada Antonio Narro 1923, Saltillo 25315, Mexico; uaaan_lacteos@yahoo.com.mx; 6 Departament of Animal Nutrition, Autonomous Agrarian University Antonio Narro, Calzada Antonio Narro 1923, Saltillo 25315, Mexico; aguileracarbo@gmail.com

**Keywords:** edible coating, food packaging, thyme oil, polymer application, guar gum coating

## Abstract

Edible coatings are safe, legal, and sensory acceptable for food applications and they can be incorporated as natural additives due to their antimicrobial activity, thickening capacity, nutrient content, and bioactive agents for protecting seafood from physical, chemical, and microbiological damage that affects its shelf-life. This study aimed to evaluate the effect of the guar gum bioactive coating with thyme oil on the quality of tilapia fish fillets for 15 days of storage at 4 °C, as a means to extend shelf-life. pH, moisture, ash, fat, color, thiobarbituric acid reactive substances (TBARS), total volatile basic nitrogen (TVB-N), microbiological, and sensory examinations were investigated, and the results were analyzed by analysis of variance. The treatments were control (uncoated, UC), GGC (coated with guar gum, GGC), and guar gum combined with thyme oil (GGCTH). Tilapia fillets were stored at 4 °C, the safe temperature for refrigerated storage for 15 days. GGCTH had a slower increase of pH after 15 days of storage in comparison with GGC and UC (*p* < 0.05). GGC and GGCTH resulted in lower and lowest lightness (L*; *p* < 0.05) values, lower and lowest redness (a*; *p* < 0.01) values, and greater and greatest yellowness (b*; *p* < 0.05) values compared to UC, respectively. UC reduced shear force at 5 (0.37 kgf), 10 (0.32 kgf), and 15 (0.30 kgf) days post-storage in comparison with GGC (0.43, 0.43, and 0.43 kgf) and GGCTH (0.43, 0.44, and 0.44 kgf), respectively. There was less (*p* < 0.05) deterioration, as well as differences in textural and sensorial variables between uncoated and coated fish fillets. The microbiological analyses demonstrated that there was greater microbial growth in the uncoated fillets than in the coated ones. It was concluded that this bioactive coating with thyme oil retards microbial colonization of fish and reduces degradability of quality variables, therefore, it is a reliable and effective alternative to extend the shelf-life of tilapia fillets.

## 1. Introduction

Nowadays, consumers have been looking for healthier foods richer in nutrients, increasing the consumption of fish and fishery products [1]. Fish is a valuable source of micronutrients, protein, essential amino acids, and lipids, which are essential for a healthy diet [2]. Tilapia (*Oreoschromis niloticus*) prospers well in tropical and subtropical waters with an adequate growth in waters with a wide range of oxygen concentration, temperature, and salinity. The global production of tilapia grew in the last decade due to its increasing acceptability and preference from consumers [3]. Tilapia fish is the second most popular species fished in Mexico, consumed mostly as fresh food, and its most frequent market presentation is fresh under refrigeration or frozen [4].

High perishability is a disadvantage of fish unlike other fresh commodities [5]. Under optimal conditions of post-capture handling, the fish products’ shelf life is mostly determined by endogenous biochemical reactions. Unfortunately, the deterioration of fatty or semi-fatty fishes does not inhibit completely with refrigeration. Fatty acids in fish are altered by the environmental oxygen which oxidizes and spoils fish meat. As a consequence, the taste becomes bitter, and color changes may occur. High water-holding capacity, neutral pH values, enzymes contained in the tissues, and lower connective tissue content have acceleratory effects on the spoiling process. During storage or processing steps, microorganisms change the protein structure and provoke unpleasant odors. All these changes alter the perceptions and satisfaction of consumers [1,5] and result in economic losses for the producers [1]. However, another reason contributing to the microbial deterioration is the poor post-harvest handling; thus, a way to delay the microbial growth is to use preservative products to increase the quality of fish and its products [4].

Few approaches have been proposed to extend the shelf life of fish meat and the preservation of nutrients of raw fish products, but it is still an incompletely investigated scientific issue [2,6]. Salt curing, the addition of chemical preservatives as butylated hydroxyanisole (BHA) and butylate hydroxytoluene (BHT) [1], active packaging, the application of edible coatings (ECs), and natural antioxidants that can protect the quality of fish meat are some alternatives against deterioration of fish meat [5,7,8].

The fresh fish shelf life can increase from five to seven days with the use of active packaging materials containing antimicrobial compounds [2]. Additionally, to active packaging, an edible coating is an efficient and eco-friendly method to maintain the quality and safety of food involving products of fish. A protective layer is formed by the edible coating, applied straight on the fish muscle to inhibit quality decline and prolong the shelf life. These coatings can be safely eaten, destroyed, or washed off during subsequent processing [2,6,8].

Coating films are developed from several materials such as lipids, proteins, and polysaccharides or combinations of these materials. Lipid-based coatings or films have proven to be excellent moisture barriers, and, when combined with proteins and polysaccharides, mechanical and barrier properties increase [9]. However, there are reports that lipid-based coatings and films affect quality characteristics of the coated food products such as appearance and gloss [10]. Another interesting application of lipid material is encapsulating bioactive compounds based on oil-in-water emulsion delivery systems in food products and has recently gained importance due to the property of the emulsions to encapsulate lipophilic bioactive ingredients reducing and preventing any degradability, controlling the administration period, and increasing bioavailability [11].

Another type of coatings and films that can be applied to food products can be made of proteins. Proteins can be fibrous or globular. Fibrous types are primary structural materials of animal tissues and water-insoluble, while the globular types are water-soluble, as well as soluble in aqueous solutions of bases, acids, and salts, and they achieve different important functions in living structures [9]. The most studied proteins as coatings and films are globular, due to their forming properties, and the interactions of the chains forming structure, which determines the stability, strength, moisture barrier, and mechanical properties. Whey protein, gelatin, soy protein, wheat gluten, corn zein, and casein have been used in the manufacture and application of edible coatings and films [9,11,12]. The development of nanostructures such as whey protein isolate nanofibers has become an important research field of study because they are considered as new packaging materials with high moisture barrier properties [12].

Polysaccharides are the most commonly given materials [4,6]. Guar Growers Mexico is a company in the north of Mexico that sows and cultivates *Cyamopsis tetragonoloba* (L.) Taub, from which guar gum (GG) is obtained [13]. Guar gum is a water-soluble polysaccharide with a linear chain of D-mannopyranose units bonded to galactose residues [13,14,15]. High availability, low cost, biodegradability, and physicochemical properties are characteristics that describe and make guar gum have multiple and important applications in food. Besides, it has similar properties as alginate, carrageenan, arabic gum, and xanthan gum, when used to make edible coatings and films, and guar gum is cheaper than any of them [13].

Generally, these films are acceptable barriers against oxygen but poor barriers against water vapor as a consequence of the hydrophilic character of polysaccharides. A way to improve the barrier properties of films or coatings has been the evaluation of various lipids such as waxes, fatty acids, and essential oils [8,16,17,18]. Due to the antioxidant and antimicrobial characteristics of the essential oils (EOs) the water barrier properties of films improve. EOs, or extracts from clove, cinnamon, rosemary, citral, oregano, lemon, orange, garlic, and thyme [19,20,21,22,23,24,25,26,27], are unquestionably the most applied ones. The important contributors to the antioxidant characteristics of oils are the volatile and nonpolar phenolic compounds [14,17,18].

A way to ensure the efficiency of the edible coatings is necessary to consider the type and characteristics of the plasticizer. The plasticizer improves the mechanical properties and the flexibility of the films, and several plasticizers are polyols [28]. A polyol widely used in the production of films and coatings based on polysaccharides is glycerol. It has properties of being polar, soluble in water, and of low molecular weight, which is why it is recommended as a plasticizer for water-soluble polymers, and it is also approved by the FDA to be used in the manufacture of polymeric coatings. The concentrations in which they are used range 0.8–75% according to the literature [29,30,31].

The objective of this research was to evaluate the influence of the bioactive coating made of guar gum (GG), glycerol (Gly), and thyme essential oil (TH) on the physical, chemical, microbiological, and sensorial changes of tilapia fillets stored at 4 °C to extend their shelf-life. There are several studies about using coatings on food products, but not as many for fish or seafood products or on using guar gum as a film or coating material combined with essential oils. The contribution of this study is to demonstrate the utility of an inexpensive, biodegradable, and antimicrobial coating based on guar gum with thyme oil as an alternative to extend the shelf-life of tilapia fillets.

## 2. Materials and Methods

### 2.1. Materials

Fresh white fillets of tilapia (*Oreochromis niloticus*) were obtained from Anahuac, a local seafood market in Saltillo, Mexico. Guar gum (GG), glycerol (Gly), and Tween-80 were purchased from Sigma-Aldrich Química, S. de RL. de CV., Mexico. Thyme essential oil was obtained from the laboratory of the Food Science and Technology Department from the Autonomous Agrarian University Antonio Narro using hydrodistillation of air-dried aerial parts of the thyme (*Thymus vulgaris*) plant. 

### 2.2. Preparation of Fish for Bioactive GG Coating Solution

Two GG coatings were prepared as follows: (a) GG solution was prepared by dissolving 1.5% of GG in distilled water with the addition of 30% glycerol (w/w GG = 0.45 g Gly/1.5 g GG per 100 mL H_2_O) in the stirring mixture (2000 rpm) and heated to 37 °C for 10 min using a stirring hot plate (Lab companion, HP-30100, Cole-Parmer, IL, USA). This formulation was used for coating fish fillets right after preparation and cooling at room temperature (20 °C). (b) GG solution was prepared by dissolving 1.5% of GG in distilled water with the addition of 30% glycerol (w/w GG) in the stirring mixture (2000 rpm). Then, 1.20% of thyme essential oil (w/w GG) and 0.90% of Tween-80 (w/w GG) were mixed in the heated solution. Solutions were homogenized using a stirring hot plate (Lab companion, HP-30100, Cole-Parmer, IL, USA) at 5000 rpm for 45 min, and right after cooling at room temperature (20 °C) were employed as a coating for fish fillets. 

### 2.3. Treatment and Storage of Fish Samples

Three treatments were evaluated in this study. The control (uncoated, UC), GG solution treatment (fillets coated with GG and glycerol, GGC), and GG combined with glycerol and thyme essential oil treatment (GGCTH). 

The tilapia fillets were cut into approximately 50 g pieces for each treatment. Uncoated samples were used as controls. Tilapia samples were then coated with GGC or GGCTH solutions using the spray coating method, letting samples stand for 30 min at 14 °C in a cool chamber, for covering the fish fillet with the coating. Samples were placed on perforated plastic trays, which were previously washed with a chlorine solution, at 4 °C in a refrigerated chamber during the 15-day study period. 

### 2.4. Physicochemical Analyses

#### 2.4.1. pH

The study was carried out from Day 0 to Day 15 of storage at 4 °C, taking samples every 5 days. pH measurements were carried out with 5 g of the sample homogenized in 45 mL distilled water. By triplicate, the pH was estimated after 5 min at 20 °C. Using a digital pH meter (Hanna Instruments, HI 98129, Italy), the pH values of samples were determined [5]. 

#### 2.4.2. Proximate Composition

AOAC official methods [32] were used to determine moisture, ash content, crude fat contents, and crude protein.

#### 2.4.3. Determination of Thiobarbituric Acid Reactive Substances (TBARS) (Lipid Oxidation)

Triplicate tilapia fillet samples (10 g) from each group were processed and analyzed, as described by Borges Vieira et al. [1]. The absorbance was measured using a spectrophotometer (Genesys 10 VIS/UV, Madison, WI, USA) at a wavelength of 532 nm, and the results were expressed in mg of malonaldehyde per kg of fish meat (mg of MDA eq. kg^−1^) [1].

#### 2.4.4. Total Volatile Basic Nitrogen Analyses (TVB-N)

The determination of TVB-N values was performed according to Kilincceker et al. [6]. The 10 g fillet samples per treatment were homogenized with perchloric acid (6%), stirred for 5 min at 3000 rpm, and then filtered through filter paper for the final titration [6].

#### 2.4.5. Color Measurements

Using a Minolta Chroma meter CR400 device (Minolta, Osaka, Japan) and the L*, a*, and b* CIELAB system, the color parameters of the tilapia fillet muscle were measured in triplicate [33]. The lightness (L*, estimates from black to white on a 0–100-point scale), redness (a*, estimates positive values corresponding to red and negative values to green), and yellowness (b*, estimates positive values for yellow and negative values for blue) were recorded [8].

#### 2.4.6. Texture Analyses

The surface-breaking force measured as the texture of the samples of tilapia fillets was estimated at a speed of 2 mm/s to cut through the sample with trigger force set at 0.2 kg m s^−2^ in a Texture Analyzer (TA-XT plus, Texture Technologies Corp., Hamilton, MA, USA), using a Warner–Bratzler cell. The shear force necessary to cut the samples is given in kgf. The surface-breaking force and the shear force were calculated from the force-time curve, applying the software package of the Texture Analyzer [34].

### 2.5. Microbiological Analyses

To assess the effectiveness of edible coating treatments in protecting fish fillets from microbial contamination, the microbial analysis was performed as follows. Approximately 10 g minced fish samples of each treatment were homogenized with 90 mL of sterile casein peptone using a stainless-steel food processor (Nutibullet series 600watt NBR-0804R, Nutribullet, LLC, Los Angeles, CA, USA) for 1 min to prepare 1:10 sample suspensions [3,35]. Serial decimal dilutions were prepared and planted onto appropriate microbiological media for detection and count of the aerobic colony, total microbial psychrotrophic, and Enterobacteriaceae, following standardized procedures [3]. The standardized procedures were executed in triplicate and means were recorded as log10 CFU/g.

### 2.6. Sensory Evaluation

For the sensory evaluation, raw fillets were analyzed using the quality index method (QIM) [5] by 15 trained panelists. For each quality attribute according to descriptions, scores ranged from 0 to 5, on Days 0, 5, 10 and 15 of storage. Tilapia received a score between fresh (QI (quality index) = 0) and completely deteriorated (QI = 5). A preference score of 3 was deemed as acceptable quality [5].

### 2.7. Statistical Analyses

The experimental design was a completely randomized model, containing three types of coating and three periods of meat evaluation, with three replications per treatment. Data were subjected to analysis of variance (ANOVA), and the results were expressed as mean ± standard deviation (SD). The treatment × time of storage interaction was included in the model. If differences existed among the sample means, differences between means were compared by the Tukey test at the level of *p* < 0.05 using the JMP software (SAS Institute Inc., Cary, NC, USA).

## 3. Results and Discussion

### 3.1. Effect of the Bioactive Coating Incorporated with Thyme Essential Oil on the pH Values of Fish Fillets

**Figure 1:**
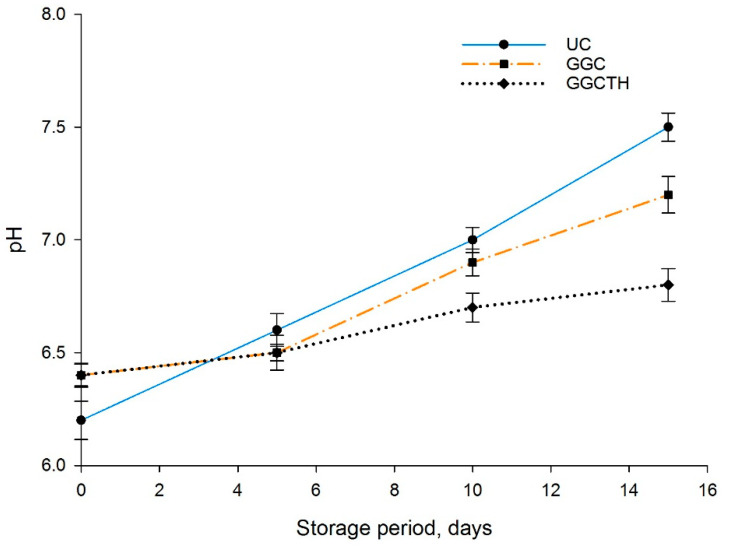
pH values variation on tilapia fillets treated with different coating methods stored at 4 °C during 15 days: uncoated sample (UC), coated with guar gum and glycerol (GGC), and coated with guar gum, glycerol, and thyme oil sample (GGCTH).

The pH values of tilapia meat subjected to treatments are shown in Figure 1. pH values showed a significant difference (*p* < 0.05) for both the storage period and treatment. The pH values were initially 6.2 and, in the case of uncoated fillets, pH increased significantly on Day 15, while GGC-treated fillets also showed an increase in pH values by Day 15. However, no significant change was observed during the storage period for GGCTH fillets. On the other hand, there was a significant difference between GGCTH and the GGC and uncoated samples. According to Cao et al. [36], Borges Viera et al. [1], Shokri et al. [7], and Umara et al. [16], the increase in pH values is related to the increase in TBV-N values, because of the action of proteolytic bacteria and autolytic enzymes capable of disintegrating chemical components containing nitrogen [4].

### 3.2. Proximate Composition

The proximate composition of tilapia fillets treated with different coating compounds and stored at refrigeration temperature (4 °C) for different periods is shown in Table 1 The proximate composition of tilapia fillets was reported by Arannilewa et al. [37], Chaparro-Hernández et al. [4], and Joukar et al. [38].

In moisture values, variations were observed, with moisture decreasing in some samples during the storage period (Table 1). An important characteristic of coating solutions that affect the quality of the fillet samples is that they inhibit moisture loss [1]. Sometimes the water permeability of the coating solution of GG is reduced [1,13,14], and a way to improve the effectiveness of this property as a barrier is to incorporate hydrophobic essential oils into the hydrophilic polymer matrix [1]. The ash content and crude fat remained unchanged throughout the storage period (15 days). For these three variables, there was no treatment × time of storage interaction (*p* > 0.25).

In Table 1, the effect of coating tilapia fillets with GGC or GGCTH on the TBARS and TVB-N variables is presented. The expressed TBARS and TVB-N values can indicate the antioxidant effect of the coating materials. Although the chemical variables’ values increased in the uncoated samples, the increase observed in the coated fillets samples (GGC and GGCTH) was slower, especially the latter coating during 15-day storage trial.

The TBARS and TVB-N values initially recorded for the uncoated and coated samples were not very different. However, at the end of the storage period (Day 15), the TBARS values were higher for uncoated samples (UC) than for coated samples (GGC and GGCTH). Therefore, the coated samples (GGC and GGCTH) were more effective in controlling deoxidation, due to the possible formation of an oxygen resistant layer on the fillets surface, hence reducing gas exchange and lipid oxidation [1,34,37,38]. The quality decrease of fish during storage is one of the main reasons for consumer rejection due to the oxidation, a non-microbial factor, of these food products [4,39,40]. The results are in agreement with data reported by Da Silva Santos et al. [34], who found lower TBARS values in chitosan-coated samples under cold temperatures. Jourkar et al. [38] reported that the TBARS values of the coated fillets with Farsi gum and cinnamon and thyme essential oils under refrigeration conditions were lower than uncoated fillets; the range of values was 2.0–5.5 mg MDA equivalents/kg of muscle for uncoated and 1.5–4.0 mg MDA equivalents/kg of muscle for coated fish. Taghi Gharibzahedi and Mohammadnabi [41] reported the values of TBARS for fish fillets coated with six treatments of jujube gum with nettle oil, which were also lower than the values for the uncoated samples during a 15-day cold-storage (4 °C) study: 0.42–3.81 mg MDA equivalents/kg of muscle for uncoated and 0.38–2.57 mg MDA equivalents/kg of muscle for coated. The treatments × time of storage interaction was significant (*p* < 0.0001), with lower TBARS in UC, compared to GGC and GGCTH at five days of storage, but a much higher decrease in TBARS in GGC and GGCTH than UC at 10 and 15 days of storage.

TVB-N has been widely used as an essential indicator of fish deterioration, and its values increase due to the autolytic and microbial deterioration that happens in the fillet, which determines the levels of nitrogen-derived compounds from different amine groups as well as ammonia [4]. The European Commission (ECC95/149) suggested TVB-N values of approximately 30–35 mg N/100 g of fish muscle at the starting point of spoilage in fresh fish. Nevertheless, several other authors have stated that 25 mg N/100 g of fish muscle is the beginning of the spoilage in the fillet of rainbow trout [36]. The increase in TVB-N values is potently related to endogenous enzyme and growth of spoilage microorganisms on fish flesh [3,15,42], thus the higher microbial counts in uncoated (control) fillet samples could characterize the behavior of the TVB-N patterns when compared with the coated samples, during the 15-day storage period (Table 2). The TVB-N values for Day 15 differed (*p* < 0.05) between treatments due to coating and the presence of thyme essential oil compared with the uncoated fillet sample. Thus, an edible coating incorporating thyme essential oil to a GG base film could improve the shelf life of fish meat by nullifying lipid oxidation and meat decomposition by microbes. These factors could have an outstanding influence on microbial growth, sensory characteristics, and shelf life of fish muscle [32]. Similar to TBARS, there was a treatment × time of storage interaction (*p* < 0.0001) for TVB-N, indicating that the effect of coatings on TVB-N depends on the storage time.

### 3.3. Color Measurements

The effect on color variables between treatments and during storage periods is presented in Table 2. On Days 5, 10, and 15 of storage, there were significant differences in L* values. However, UC treatment showed a significant increase (*p* < 0.05) in L* values compared to GGC, while GGCTH remained almost unchanged during storage, but it was significantly darker than the UC fillet. Higher L*values denoted more transparency, luminosity, and brightness; the reason for this change in reflectance is probably due to protein oxidation [4]. The values for a* showed differences (*p* < 0.05) between GGC and GGCTH treatments, as well as for different storage times, possibly as a result of the presence of the thyme oil in GGCTH. The b* values of UC, GGC, and GGCTH differed (*p* < 0.05) for both storage periods and treatments. These characteristics may be explained by the presence of components of the bioactive coating applied to the tilapia fillets [34]. According to the hue circle, all treatments during the storage time of the tilapia fillets were within the yellow-red zone (data not shown).

### 3.4. Texture Analysis

**Figure 2:**
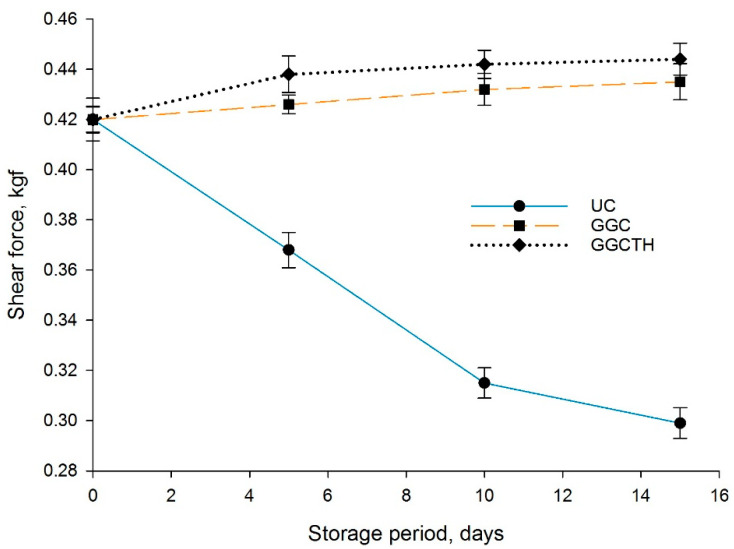
Influence of coating on shear force of tilapia fillets stored at 4 °C during a 15-day trial: uncoated sample (UC), coated with guar gum and glycerol (GGC), and coated with guar gum, glycerol, and thyme oil sample (GGCTH).

For consumer acceptability of any fishery product, texture is an important factor. After fish are slaughtered [8] and then progress during low-temperature storage, the texture is affected by autolytic and microbiological processes, as well as the intrinsic factors such as protein, lipids, and collagen contents. Among the texture factors describing the quality of fish is hardness, measured by the shear force [41]. The texture of tilapia fillets was analyzed by shear force (kgf) on Days 5, 10, and 15 (Figure 2). The lowest value on Day 5 was found in the texture of UC fillets, while coated fillets presented a firmer texture of muscle fibers with differences (*p* < 0.05) among treatments. As shown in Figure 2, the shear force values for the UC fillets were lower than those for GGC and GGCTH on Days 10 and 15. Chaparro-Hernández et al. [4], Taghi Gharibzahedi and Mohammadnabi [41], and Saéz et al. [8] revealed that the death of fish starts autolysis, causing the muscle to be softer and less elastic, and this procedure increases by microbial participation. Because the storability and shelf life of fish can be determined by the flesh softening and texture changes during storage, the use of bioactive coatings enriched with thyme essential oil can guarantee lower deterioration rate due to oxidative rancidity. A way to guarantee a reduction in deterioration rate due to oxidation and avoid flesh softening and texture changes during storage is the application of a bioactive coating with thyme essential oil.

### 3.5. Impact of Coating on the Microbiological Quality of Tilapia Fillets

**Figure 3:**
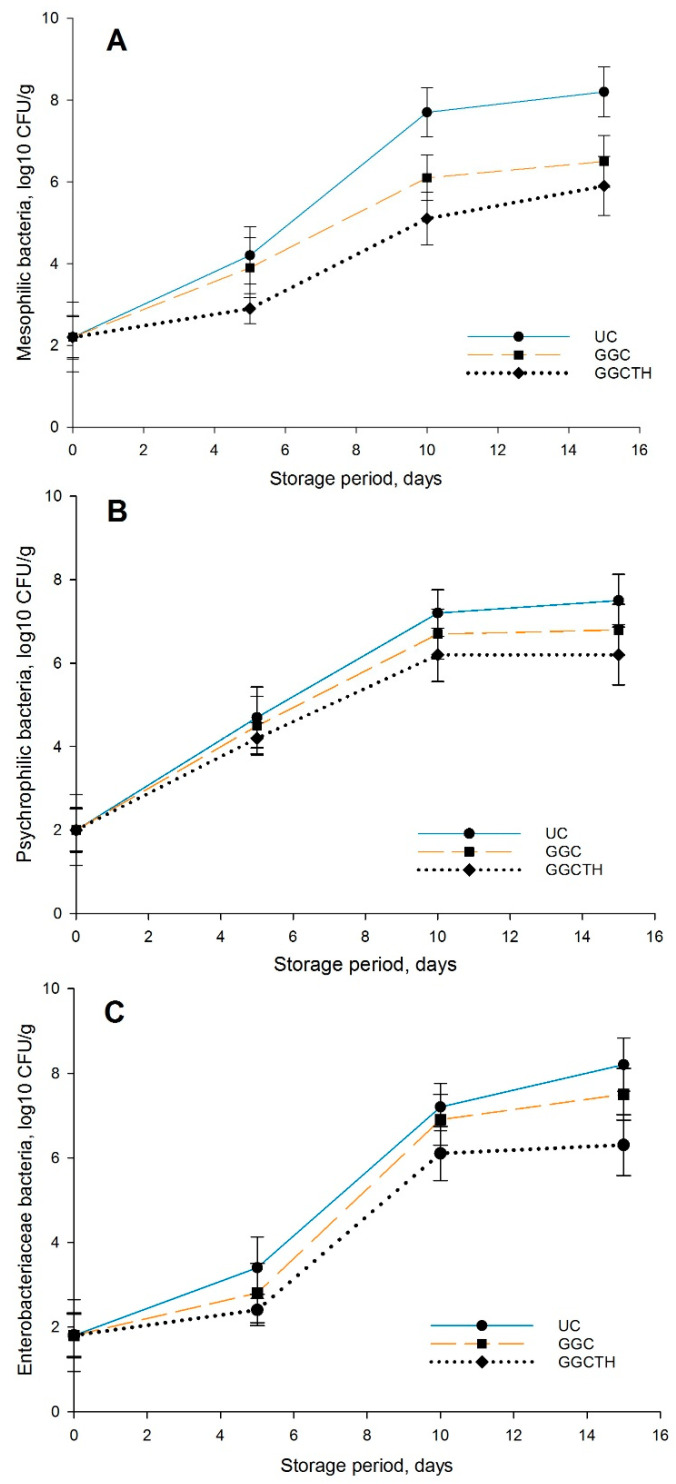
Effect of coating on the microbial growth were (**A**) corresponds to mesophilic bacteria, (**B**) to Psychrophilic bacteria, and (**C**) to Enterobacteriaceae bacteria, in the tilapia fillets samples stored at 4 °C: uncoated (UC), coated with guar gum and glycerol (GGC), and coated with guar gum, glycerol, and thyme oil (GGCTH).

Global food production is affected by microbial spoilage, due to the action of various microorganisms responsible for the spoilage of fresh and poorly preserved seafood. The effect of UC, GGC, and GGCTH on the growth of mesophilic (MBC), psychrophilic (PBC), and Enterobacteriaceae bacteria (EBC) during storage time at 4 °C until Day 15 is shown in Figure 3. The initial mesophilic bacteria count (log10 CFU/g) in all samples of tilapia fillet was 2.00 log10 CFU/g, indicating a good quality of fish meat. The initial value of the psychrophilic bacteria count of fresh fillets was 2.00 log10 CFU/g. MBC, PBC, and EBC of all samples increased gradually as the storage period increased (*p* < 0.05) (Figure 3A–C). By Day 10 of storage, MBC in tilapia fillets reached 7.7 log10 CFU/g for UC samples, which is higher than the recommended value in raw fish [36,38], showing a shelf life of about nine days for the UC treatment. For GGC, the value was 6.1 log10 CFU/g, and, for GGCTH, it was 5.1 log10 CFU/g (Figure 3A). At the end of the storage time (Day 15), the guar gum and thyme oil coatings showed MBC values that did not exceed the maximal permissible limit (5.9 log10 CFU/g). All of the coated treatments led to a significant reduction in MBC, PBC, and EBC in tilapia fillet compared to the UC fish meat. Jouki et al. [43], Joukar et al. [38], and Coa et al. [36] mentioned values for total viable counts for tilapia, trout, and silver perch around 2–6 log10 CFU/g. Jouki et al. [43] and Volpe et al. [24] reported initial total viable counts around 4.5 log10 CFU/g and an increase in the total viable count during storage. The bacterial growth in coated samples containing essential oils was less than in uncoated samples. The antibacterial effects of natural essential oils may contribute to these differences [38]. Lee et al. [44] also mentioned that the control had no antimicrobial effect on the total viable count, but the skate skin gelatin (SSG) film with some percentage of thyme essential oil had lower values than the control samples. 

Among the microorganisms responsible for the deterioration of fresh fish are Enterobacteriaceae, which are considered an indicator of hygiene, because it is part of the microflora of the fish as well as the microorganisms that are acquired during the processing of fresh meat [45]. The initial value of Enterobacteriaceae was 1.8 log10 CFU/g for the three treatments, which is lower than the initial value of rainbow trout fillets (2.27 log10 CFU/g) reported by Ozogul et al. [46], but these values reached 8.2, 7.5, and 6.3 log10 CFU/g in the UC, GGC, and GGCTH, respectively, at the end of the storage period (Figure 3A–C). Oz [47] reported that coating of fish fillets, either with gelatin-based film solely or in combination with garlic essential oil, led to inhibition of Enterobacteriaceae growth during the storage, reaching values of 7.01, 6.93, and 6.48 log10 CFU/g in the three fillet samples. Oz [47] concluded that the addition of garlic into the rainbow trout diet reduced the number of Enterobacteriaceae in fish muscle maintained at cold temperatures during storage.

The use of GGC avoided the increase of microbial count and provided a nonstop decrease in these counts along with the storage at 4 °C compared to the uncoated fillets, due to the barrier properties inherent to the GG as a coating agent. Furthermore, the addition of thyme essential oil to the GGCTH film strengthened its antimicrobial activity against all the studied microorganisms.

### 3.6. Influence of Coating on the Sensory Properties of Tilapia Fillets

**Figure 4:**
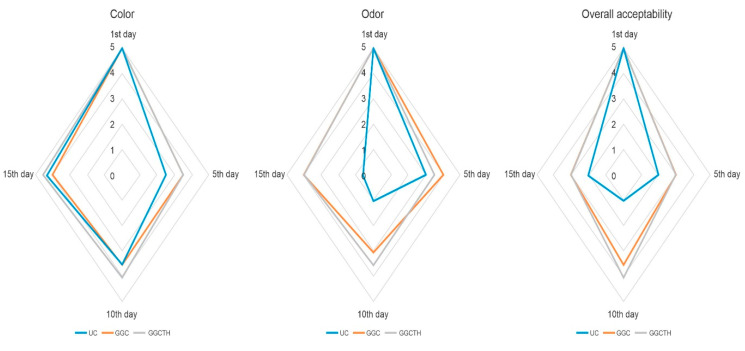
Changes in the sensory quality attributes of color, odor, and overall acceptability of tilapia fillets samples stored at 4 °C 15 days: uncoated (UC), coated with guar gum and glycerol (GGC), and coated with guar gum, glycerol, and thyme oil.

Changes in the sensory attributes of color, odor, and overall acceptability of the fillet samples are shown in Figure 4. The scores are given by the panelists for each attribute according to the descriptions ranging from QI = 1 for the lowest acceptability or preference to QI = 5, corresponding to the highest acceptability or preference score [5]. On Day 1, all fillet samples had a score of 5 for the three attributes evaluated; as the days passed, the scores decreased (*p* < 0.05) for UC fillets; however, this tendency did not show up with the coated tilapia fillet samples. The changes in scores on GGC and GGCTH were less perceptible (*p* < 0.05) than the UC, and the coating incorporating thyme essential oil obtained higher scores than coating without it. Fillets with scores below 3 were considered unacceptable due to putrid odor, opaque color, and overall unacceptability. On Day 10 of storage, some spoilage signs were noticed by the panelists, because of the off-odor and off-color of the UC samples, which were unaccepted after that day of storage (score = 1). Nevertheless, GGCTH samples obtained higher scores than GGC during storage time. The incorporation of essential oils on coatings with antioxidant and antimicrobial compounds has shown a positive effect to extend the shelf life of fish at 4 °C to approximately 12 days with fish gelatin coating and oregano EOs on rainbow trout fillets [48], 15 days with carrageenan coating with lemon EOs on trout fillets [24], 16 days with sodium alginate coating with horsemint EOs on bighead carp fillets [49], 16 days with carboxymethylcellulose coating with *Pimpinella affinis* EOs on silver carp fillets [50], and 20 days with carboxymethylcellulose coating with *Zataria multiflora Bois* EOs on trout fillets [51]. The results of the sensory evaluation were in agreement with those of TVB-N and microbiological analyses.

## 4. Conclusions

The results of the present work show that tilapia fillets coated with guar gum, glycerol, and thyme oil may effectively retard lipid oxidation and protein deterioration and delay the growth of mesophilic bacteria, psychrophilic bacteria, and Enterobacteriaceae. The application of the guar gum, glycerol, and thyme oil coating improved physical and sensorial characteristics within acceptable scores throughout the storage and may prolong the shelf life of tilapia fillets to 15 days compared to the uncoated fish. Therefore, the guar gum, glycerol, and thyme oil coating may be recommended for the extension of tilapia fillets shelf life, preserving the physical, chemical, microbiological, and sensory quality by the use of a reliable, safe, and natural preserving agent.

## Figures and Tables

**Table 1:** Physicochemical properties of tilapia fillet samples determined during a 15-day trial stored at 4 °C.

Physicochemical Variables	Storage Period (Days)	Treatments		
		UC	GGC	GGCTH
Moisture, %	0	89.95 ± 0.33 ^aB^	90.02 ± 0.25 ^aAB^	90.01 ± 0.18 ^aA^
	5	82.65 ± 0.75 ^bB^	83.41 ± 0.42 ^bAB^	84.17 ± 0.28 ^bA^
	10	74.16 ± 0.53 ^cB^	75.31 ± 0.03 ^cAB^	76.46 ± 0.53 ^cA^
	15	62.19 ± 0.93 ^dB^	62.97 ± 0.33 ^dAB^	63.76 ± 0.04 ^dA^
Ash content, %	0	1.69 ± 0.03 ^aA^	1.70 ± 0.04 ^aA^	1.68 ± 0.05 ^aA^
	5	1.75 ± 0.01 ^aA^	1.85 ± 0.01 ^aA^	1.96 ± 0.02 ^aA^
	10	2.04 ± 0.01 ^aA^	1.99 ± 0.01 ^aA^	1.95 ± 0.03 ^aA^
	15	2.11 ± 0.21 ^aA^	1.99 ± 0.01 ^aA^	1.88 ± 0.05 ^aA^
Crude fat, %	0	1.47 ± 0.57 ^aA^	1.47 ± 0.25 ^aA^	1.48 ± 0.42 ^aA^
	5	1.87 ± 0.58 ^aA^	1.60 ± 0.17 ^aA^	1.51 ± 0.20 ^aA^
	10	1.72 ± 0.03 ^aA^	1.64 ± 0.01 ^aA^	1.70 ± 0.01 ^aA^
	15	1.66 ± 0.03 ^aA^	1.83 ± 0.02 ^aA^	1.87 ± 0.04 ^aA^
TBARS, mg of malonaldehyde/kg fillet	0	0.17 ± 0.22 ^dA^	0.19 ± 0.60 ^dA^	0.18 ± 0.11 ^dA^
	5	0.20 ± 0.65 ^cB^	0.23 ± 0.01 ^cA^	0.23 ± 0.10 ^cA^
	10	0.39 ± 0.18 ^bA^	0.28 ± 0.24 ^bB^	0.28 ± 0.17 ^bB^
	15	0.54 ± 0.90 ^aA^	0.31 ± 0.65 ^aB^	0.30 ± 0.03 ^aB^
TVB-N, mg N/100 g fillet	0	16.03 ± 0.32 ^dA^	16.04 ± 0.20 ^dA^	15.99 ± 0.47 ^dA^
	5	27.70 ± 0.65 ^cA^	19.56 ± 0.01 ^cB^	16.44 ± 0.10 ^cC^
	10	37.68 ± 0.18 ^bA^	26.04 ± 0.24 ^bB^	19.72 ± 0.17 ^bC^
	15	51.32 ± 0.90 ^aA^	29.24 ± 0.65 ^aB^	22.03 ± 0.03 ^aC^

UC, uncoated tilapia fillet; GGC, coated tilapia fillet with guar gum and glycerol coating; GGCTH, coated tilapia fillet with guar gum, glycerol, and thyme essential oil. For treatment, means with different uppercase letters in the same row differ (*p* < 0.05). For storage time, means with different lowercase letters in the same column differ (*p* < 0.05).

**Table 2:** Color variable differences of uncoated and coated tilapia fillet samples during 15-day storage at 4 °C.

Color Variables	Storage Period (Days)	Treatments		
		UC	GGC	GGCTH
L*	0	52.80 ± 0.23 ^cA^	51.38 ± 0.48 ^bB^	49.62 ± 0.49 ^bC^
	5	52.95 ± 0.34 ^cA^	51.54 ± 0.22 ^bB^	50.12 ± 0.76 ^aC^
	10	53.10 ± 0.25 ^bA^	49.91 ± 0.45 ^cB^	46.71 ± 0.65 ^cC^
	15	55.70 ± 0.68 ^aA^	52.84 ± 0.28 ^aB^	49.97 ± 0.39 ^bC^
a*	0	−0.78 ± 0.82 ^dA^	−0.59 ± 0.52 ^cAB^	−0.45 ± 0.61 ^cB^
	5	−0.84 ± 0.13 ^cA^	−0.65 ± 0.81 ^cAB^	−0.46 ± 0.26 ^cB^
	10	2.36 ± 0.87 ^aB^	3.66 ± 0.35 ^aAB^	4.96 ± 0.61 ^aA^
	15	1.33 ± 0.41 ^bA^	1.15 ± 0.76 ^bAB^	0.96 ± 0.74 ^bB^
b*	0	2.75 ± 0.24 ^bA^	1.99 ± 0.50 ^bB^	0.97 ± 0.23 ^bB^
	5	2.94 ± 0.58 ^bA^	2.01 ± 0.27 ^bB^	1.07 ± 0.51 ^bB^
	10	5.22 ± 0.33 ^aB^	6.77 ± 0.60 ^aA^	6.32 ± 0.43 ^aA^
	15	2.61 ± 0.52 ^bB^	7.79 ± 0.42 ^aA^	7.97 ± 0.55 ^aA^

UC, uncoated tilapia fillet; GGC, coated tilapia fillet with guar gum and glycerol coating; GGCTH, coated tilapia fillet with guar gum, glycerol, and thyme essential oil. For treatments, means with different uppercase letters in the same row differ (*p* < 0.05). For storage period, means with different lowercase letters in the same column differ (*p* < 0.05).
